# The role of IAEA in preparation of recommendations for the use of PET/CT in radiotherapy planning

**DOI:** 10.2349/biij.3.1.e19

**Published:** 2007-01-01

**Authors:** I Stojkovski, M Dondi, B Jeremic, P Andreo

**Affiliations:** Division of Human Health, Department of Nuclear Sciences, International Atomic Energy Agency, Vienna, Austria

The Department of Nuclear Sciences and Applications (NA) of the International Atomic Energy Agency (IAEA) is committed to contribute to sustainable development in Member States through the use of nuclear sciences and their applications in several fields, including human health. The Division of Human Health (NAHU) executes its programme’s activities through four sections: Applied Radiation Biology and Radiotherapy (ARBR), Nuclear Medicine (NM), Dosimetry and Medical Radiation Physics, and Nutritional and Health-Related Environmental Studies.

The objective of the IAEA programme in Human Health is to enhance the capabilities in Member States to address needs related to the prevention, diagnosis and treatment of health problems through the application of nuclear techniques. In particular, ARBR deals primarily with the clinical (medical) use of radiation for the treatment of diseases, mostly cancer. The main objective is to improve the availability and safe use of effective cancer management strategies in Member States, in particular by helping establish new treatment centres in countries lacking the basic internationally-accepted level of treatment and establishing resource-sparing treatment protocols, including guidelines for the treatment of the most common cancers in the limited-resource setting. The section also organises teaching events and training courses for radiation oncologists and support staff, and develops more effective treatments for different types of cancer based on radiobiological principles, and clinical and laboratory studies. Finally, the section also organises consultant and technical meetings for which it uses experts from different countries to discuss current aspects in the field of radiation oncology and radiobiology of particular importance for member states and uses these meetings to advise member states of the appropriate implementation in daily practice. Using the same means, the mission of NM is to enhance capabilities of developing Member States to address important health needs by the use of nuclear medicine functional studies or molecular biology techniques in several types of disease, including cancer. In this latter field, among other activities NM supports the implementation of PET programs in countries where national priorities and cancer management programs include that technology.

Indeed, its already widely accepted biomedical techniques involving some type of radiation are often the sole means of diagnosis and treatment in a large number of health problems, or complement non-nuclear techniques. Radiotherapy, one of the earliest applications of radiation, remains a major cost-effective modality available for cancer treatment, often in conjunction with diagnostic radiology and nuclear medicine procedures for tumour localisation.

In order to optimise diagnostic and treatment approaches to cancer conditions using nuclear technology, NAHU acts as a multitask division, frequently identifying suitable topics and areas of oncology where cross-cutting activities of several sections are identified and discussed. Some of the most recent cross-cutting activities in NAHU included consultant meeting in optimisation of palliation of bone metastasis (December 2005), a technical meeting on intensity-modulated radiation therapy (IMRT) (June, 2006) and a consultant meeting on nutrition and cancer (October 2006).

In relation to cancer management, one of the fastest growing techniques in developed countries is PET with increasing involvement of nuclear medicine in the domain of oncology, including radiation oncology. Functional imaging by means of PET has facilitated the evaluation of tumour physiology, metabolism and proliferation, which are parameters relevant to the outcome following treatment. It has been proved that compared with CT, PET has higher sensitivity and specificity for staging many types of cancer, higher sensitivity and specificity for imaging their recurrences, and higher sensitivity and specificity for monitoring the effects of therapy.

For radiotherapy planning (RTP), CT represents the current gold standard. However, the development of biologic imaging by PET brings new information about cancer. The modern images integrate many biologic aspects, including data on genotypic and phenotypic expression, metabolic activity, and cell proliferation. These elements are very useful to draw more specific strategies for treating cancer. Nuclear medicine with PET offers significant contributions in defining the volume for treatment. This imaging is based on different radiopharmaceuticals, and is able to describe glucose metabolism, cell proliferation, angiogenesis, and/or hypoxia. The nuclear medicine images can be co-registered and fused with CT and also MRI, and give both morphological and functional information that are incorporated into RTP for a better definition of the target volumes.

The integration of nuclear medicine imaging into RTP leads to a definition of a new concept of target, the biological target volume (BTV). According to the radiopharmaceutical used, the BTV expresses different properties of the tumour. The term of ‘theragnostic imaging’ as the multimodality imaging can be kept as a guide for designing the highest dose spots in the context of the tumour. The difference between this concept and the morphological imaging is that it provides information to determine how and not only where radiation therapy should be delivered. The attention should also be focused on the functional aspects and the importance of healthy tissues so as to reduce the radio-induced damage.

The inclusion of flourodeoxyglucose (FDG)-PET in the RTP has as first goal, the improvement of cancer staging and the identification of the tumour volume, using the anatomic imaging as a frame for the calculation of the dose. This may have significant effects. The impact of PET on the target delineation is related to the accuracy of the study (diagnostic sensitivity and specificity) for the type of cancer to be treated. PET affects the treatment planning according to the requests from the radiation oncologists.

Besides the added value in defining planning target volume (PTV), the most promising use of multimodality imaging is the characterisation of biochemical and physiological features of the tumour, and this can guide the delineation of tumour sub-volumes to be boosted.

A further possibility offered by the metabolic imaging is the prediction of the responsiveness to radiotherapy during the early phase of treatment. Monitoring the reduction of FDG uptake (through SUV changes) in a semi-quantitative way, can allow the optimisation of the treatment and modify the strategy of the original plan. Another role of nuclear medicine in RTP is the definition of healthy tissues to be spared, instead of the tumour volumes delineation. Imaging can allow the identification of non-functional tissues and thus guide the beam set-up.

The use of multimodal imaging in the RTP requires several steps. First of all, the image fusion that is the transfer of information from one study to another. The second step is the image co-registration, which gives a spatial mapping of the corresponding points of the images. The third step is the delineation of the volume based on a metabolic radiopharmaceutical uptake.

Different positron-emitting radionuclides must be used and their pharmacokinetics optimised before implementing PET in routine clinical practice for the purpose of changing radiotherapy (RT) volumes and predicting response to RT. Moreover, attention needs to be focused on patient motion, partial volume effects and image resolution, in order to decrease artefacts and improve image quality. Therefore, it should be understood that the use of PET for target volumes delineation requires specific parameters for image acquisition and processing, and these parameters may differ according to the different tumour types. For instance, there are several ways to assess tumour viability by quantifying tracer uptake in the tumour and to project this information onto the anatomical transaxial CT images. In fact, although the nuclear physician makes the analysis mostly with a qualitative approach, the radiation oncologist needs a quantitative evaluation. This is an emerging field, but to date robust clinical data are still needed.

Specialists in radiation oncology, nuclear medicine, diagnostic radiology and medical physicists are involved in this work, where appropriate team coordination is crucial for effective application of the technology. One of IAEA’s projects is to work on the standardisation of PET/CT protocols across the different centres throughout the world.

A consultant meeting was held in Vienna in July 2006 to advise the IAEA on the current state of the art use of PET/CT in the treatment planning of the most common solid tumours, with special emphasis on lung cancer. This consultant meeting was the basis for discussions and advice to the IAEA on the suitability of having a Coordinated Research Project (CRP), which will include participation of scientific investigators from Member States, and also to discuss and draft a manuscript/review article summarising the above. During the consultant meeting, participants presented the institutional experience in this field. The relevant literature was reviewed and the areas of major interest and research identified. Also, the implementation of this technology in developing countries had been thoroughly discussed from both clinical research and cost-effectiveness standpoints. Topics for CRPs in developing countries were identified. This activity may have special interest for centres starting to use PET/CT in RTP in developing countries.

It was unequivocally agreed that IAEA should further assess and eventually promote the usage of PET/CT in RTP. Standardisation of the techniques to delineate target volumes with PET/CT was seen as essential and this issue needs to be developed. Clinical outcome after implementation of such a technique in RT should be assessed in different tumour types in order to justify its use. Conducting multicentric cooperative international studies should be promoted to help accomplish these objectives in the near future.

Major recommendations from this meeting were that IAEA should consider providing more developing countries with three-dimensional conformal radiation therapy (3D-CRT) technology to make them adequately equipped for the introduction of PET-CT in the treatment planning process. An increasing knowledge-transfer of PET-CT technology to developing countries should be sought, providing appropriate nuclear medicine infrastructure and adequate human resources where this is available. Research efforts on the integration of PET imaging into RTP should be encouraged, providing appropriate 3D-CRT infrastructure and adequate human resources. To obtain sustainable quality in this novel approach, standardisation of PET and PET/CT acquisition protocols for both staging and treatment planning, and also PET/CT-based target volume delineation for RTP should be encouraged.

Using IAEA-funded CRP framework, timely activities should be concentrated on the evaluation of outcome of PET-based RTP, more likely in non-small cell lung cancer due to the overall burden of these tumours worldwide. Last, but not least, IAEA should encourage the clinical evaluation of new radiopharmaceuticals to aid in the delineation and the biological characterisation of the gross tumour volume.

It was proposed that IAEA consider the following ideas as suitable for potential CRPs: standardisation of delineation of target volumes using PET-based treatment planning; evaluation of local failure after either CT-based or PET-based radiation therapy; changes in PET scans after radiation therapy as a predictor of response and determination of salvage treatment.

The consultant meeting was proven to be a fruitful event enabling IAEA/NAHU to continue monitoring current achievements in this field and to put emphasis on research studies aimed at evaluating the use of PET-CT in RTP, a task to be defined during a planned meeting in 2007. To further strengthen its interest in the application of this technology in monitoring the treatment response in cancer patients, ARBR and NM are planning to organise another consultant meeting in 2007 as one of the possible activities that may request timely IAEA-funded research in this field.

